# Mitochondrial DNA diversity and demographic history of Black-boned chickens in China

**DOI:** 10.1080/23802359.2021.1912668

**Published:** 2021-04-23

**Authors:** Xunhe Huang, Zhuoxian Weng, Yujing He, Yongwang Miao, Wei Luo, Xiquan Zhang, Fusheng Zhong, Bingwang Du

**Affiliations:** a Guangdong Provincial Key Laboratory of Conservation and Precision Utilization of Characteristic Agricultural Resources in Mountainous Areas, Guangdong Innovation Centre for Science and Technology of Wuhua Yellow Chicken, School of Life Science, JiaYing University, Meizhou, China; b College of Animal Science and Technology, Hunan Agricultural University, Changsha, China; c Faculty of Animal Science and Technology, Yunnan Agricultural University, Kunming, China; d College of Animal Science, South China Agricultural University, Guangzhou, China

**Keywords:** Genetic diversity, Black-boned chicken, mtDNA d-loop, haplogroup, breeding history

## Abstract

Black-boned chickens (*Gallus domesticus*, herein abbreviated BBCs) are well known for their unique appearance and medicinal properties and have a long breeding history in China. However, the genetic diversity and demographic history of BBCs remain unclear. In this study, we analyzed 844 mitochondrial DNA D-loop sequences, including 346 *de novo* sequences and 498 previously published sequences from 20 BBC breeds. We detected a generally high level of genetic diversity among the BBCs, with average haplotype and nucleotide diversities of 0.917 ± 0.0049 and 0.01422, respectively. Nucleotide diversity was highest in populations from Southwest China (0.01549 ± 0.00026), particularly in Yunnan Province (0.01624 ± 0.00025). Significant genetic divergence was detected between most breeds, particularly between Yunnan chickens and those from all other provinces. Haplogroups F and G had the highest levels of genetic diversity and were restricted to Southwest China, particularly Yunnan Province. Based on neutrality tests and mismatch distribution analyses, we did not obtain evidence for rapid population expansions and observed similar demographic histories in BBCs and local non-BBCs. Our results suggest that Chinese BBCs have complex breeding histories and may be selected *in situ* from local domestic chickens. These results improve our understanding of the genetic heritage and breeding histories of these desirable chickens.

## Introduction

Black-boned chickens (*Gallus gallus domesticus*; herein abbreviated BBCs), renowned for their characteristic black skin, bone, and muscle, have a long history, appearing in the ancient Chinese herbology volume “Bencao Gangmu” written around 1578 C.E. (Li 2005). In China, over 18 BBC breeds have been recorded across 11 provinces (China National Commission of Animal Genetic Resources 2011). For example, Silkie, described in the thirteenth century travelogue “The Travels of Marco Polo” (Benedetto 2014), is a famous breed defined by a set of ten traits: walnut-shaped comb, dark wattles, turquoise-blue earlobes, bearded, silky feathers, five toes, black skin, bones, and meat, and booted feet (China National Commission of Animal Genetic Resources 2011). Studies of BBCs have focused on the chemical properties of the meat (Jaturasitha et al. 2008; Tian et al. 2011), distribution of melanin pigmentation (Nganvongpanit et al. 2020), molecular mechanism underlying melanin deposition (Dorshorst et al. 2011; Shinomiya et al. 2012; Yu et al. 2018; Li et al. 2019), as well as the origin and evolution of fibromelanosis (Dharmayanthi et al. 2017; Sohn et al. 2018). Additionally, genetic analyses based on genetic markers, such as microsatellites (Tang et al. 2005; Qu et al. 2006; Yu et al. 2006) and mitochondrial DNA (mtDNA) (Zhu et al. 2014; Guo et al. 2017; Jia et al. 2017; Zhang et al. 2018; Liu et al. 2018; Weng et al. 2019) have attempted to unravel the population genetic history of BBCs. However, the scarcity of sampled breeds is a major limitation of these studies, and this issue is further compounded by the complexity of chicken demographics and domestication histories (Tixier-Boichard et al. 2011; Miao et al. 2013; Lan et al. 2017; Huang et al. 2018a). Consequently, the patterns of genetic diversity and population history of BBCs, including the degree of similarity in demographic trajectories among breeds endemic to different regions, remain unclear.

Since the 1990s, mtDNA D-loop has been widely used to trace the history of chicken domestication owing to its high mutation rate, lack of recombination, and maternal inheritance (Di Lorenzo et al. 2015; Lan et al. 2017). In the current study, we evaluated an extensive mtDNA D-loop dataset for various BBC breeds spanning a wide geographical distribution to reassess genetic diversity and divergence across China. Additionally, a d-loop dataset for local non-BBC populations was used for comparative analyses to infer the demographic histories of BBCs.

## Materials and methods

### Sample collection

A total of 346 wing-vein blood samples were collected and stored at −80 °C in the sample database of Jiaying University. These samples were obtained from 13 BBC breeds distributed across nine provinces in China (Table S1). Animal handling and experimentation followed the animal experimental procedures and guidelines approved by the Ethics Committee of Jiaying University (#20151103). Genomic DNA was extracted using the standard phenol–chloroform method, and the DNA concentration and purity were assessed by gel electrophoresis and using a NanoDrop Spectrophotometer 2000 (ThermoFisher Scientific, Waltham, MA). Additionally, 498 published mtDNA d-loop sequences for 15 BBC breeds in China (Table S2) as well as mtDNA d-loop sequences of local non-BBCs (Table S3) were retrieved from GenBank (www.ncbi.nlm.nih.gov).

### PCR amplification and sequencing

The d-loop was amplified using the primers L16750 (5′-AGGACTACGGCTTGAAAAGC-3′′) (Fumihito et al. 1994) and H522 (5′-ATGTGCCTGACCGAGGAACCAG-3′′) (Fu et al. 2001). PCR amplifications were performed using a Bio-Rad thermal cycler (Bio-Rad, Hercules, CA) in a final volume of 30 μL containing 3 μL of 10× buffer (Mg^2+^), 2.4 μL of dNTPs (2.5 mM), 0.3 μL of each primer (20 pmol/μL), 0.3 μL of rTaq polymerase (5 units/μL; TaKaRa, Berkeley, CA), 23.2 μL of ddH_2_O, and 0.5 μL of genomic DNA (50–100 ng/μL). The cycling profile included a 4-min preliminary denaturation cycle at 94 °C, followed by 35 cycles of denaturation at 94 °C for 30 s, annealing at 63 °C for 1 min, and extension at 72 °C for 50 s, with a final extension at 72 °C for 10 min.

The PCR products were separated by electrophoresis on a 1.5% agarose gel containing GelRed (Biotium Inc., Fremont, CA), and visualized under ultraviolet light. Sequencing of the D-loop was carried out at Guangzhou IGE Biotechnology Co., Ltd. (Guangzhou, China) using an ABI 3730xl Analyzer (Applied Biosystems, Foster, CA).

### Data analyses

DNA sequences were manually checked using BioEdit (Hall 1999), and then aligned using the ClustalW algorithm in MEGA 6.0 (Tamura et al. 2013). All sequences were aligned and trimmed to 518 bp, corresponding to nucleotide positions (nps) 1–518 of the red junglefowl reference sequence AP003321. Haplotypes were defined using DnaSP 6.10.01 (Rozas et al. 2017). These sequences were then assigned to specific haplogroups according to DomeTree (Peng et al. 2015a). Nucleotide (*π*) and haplotype diversities (*Hd*) were calculated using DnaSP 6.10.01 (Rozas et al. 2017). A median-joining network of d-loop sequences was constructed using NETWORK 5.0 (Bandelt et al. 1999). To assess population genetic differentiation and gene flow among sampling locations, pairwise *F*_ST_ values were computed using ARLEQUIN 3.5 with 10,000 permutations (Excoffier and Lischer 2010). These pairwise *F*_ST_ values were then used to generate non-metric multidimensional scaling plots using SPSS 19.0 (IBM Corp., Armonk, NY). To further explore geographical structure, an analysis of molecular variance (AMOVA) was also calculated in ARLEQUIN 3.5 (Excoffier and Lischer 2010) using breeds (populations), provinces or regions as groups. A mismatch distribution analysis with the expectations of a sudden expansion model was performed and population expansion statistics (Fu’s *Fs* and Tajima’s *D*) were calculated using ARLEQUIN 3.5 (Excoffier and Lischer 2010) with 10,000 permutations.

## Results

### Geographical distribution of mitochondrial haplogroups

Our analysis included 844 mtDNA D-loop sequences (346 *de novo*; GenBank accession numbers: MH923578–MH923923) belonging to 20 BBC breeds in China. In total, 116 haplotypes were identified (nps 1–518), including 90 haplotypes exclusive to a single breed and 26 haplotypes shared by two or more breeds. Yunnan Province had the most haplotypes (54), followed by Zhejiang (33), Guizhou (24), and Sichuan (15). BBCs from Yunnan and Zhejiang provinces had more private haplotypes (Table S4). The 116 haplotypes belonged to haplogroups A–G and Z (Table S4). The geographical distribution of mtDNA haplogroups of BBCs is shown in Table S5. Interestingly, a similar pattern (haplogroups F and G) was also observed in non-BBCs in China (Table S6). Overall, all seven haplogroups were found in Yunnan Province, whereas the other provinces lacked two or more haplogroups.

**Figure 1.**
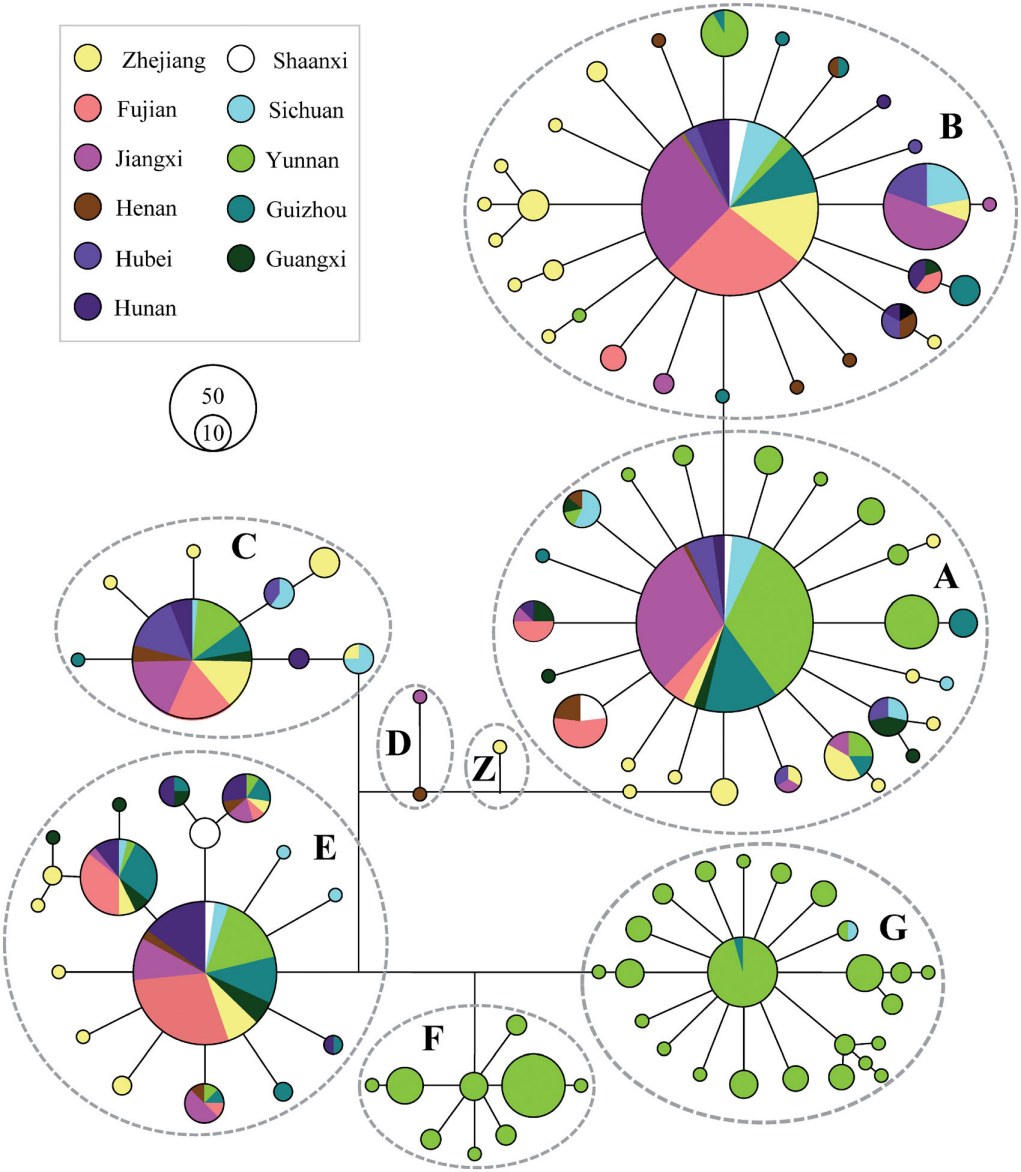
Median-joining network of mtDNA haplotypes of BBCs from eleven provinces. The links are labeled by the nucleotide positions to designate transitions. Cycle sizes are roughly proportional to the haplotype frequency.

A median-joining phylogenetic network based on 844 mtDNA D-loop sequences showed a clear geographical pattern (Figure 1). Haplogroups A, B, C, E, and G formed star-like patterns in the network, each of which had a predominant haplotype and few derived haplotypes. Haplogroups D and Z were rare, only occurring in Henan, Jiangxi, and Zhejiang Province. Haplogroup F, detected only in Yunnan Province, was found at a particularly high frequency in the Tengchong white breed, while haplogroup G was mainly observed in the Yanjin Black-boned breed, and only one haplotype was detected in each of Sichuan Silky and Sichuan Mountain Black-boned breeds. The median-joining network of mtDNA d-loop sequences for BBCs (*n* = 844) and local non-BBCs (*n* = 1663) did not demonstrate BBC-specific or non-BBC-specific patterns, and the two groups shared the same haplogroups or major haplotypes (Figure S1).

### Genetic diversity

The *Hd* and *π* values, and the average number of nucleotide differences (*K*) for all samples were 0.917 ± 0.0049, 0.01442, and 7.365, respectively (Table 1). The Wuliangshan Black-boned breed had the highest *π* value (0.01633 ± 0.00048), while the Silkies breed had the lowest (0.00733 ± 0.00063). With respect to the 11 provinces, BBCs from Yunnan Province had the highest *π* value (0.01624 ± 0.00025), while those from Jiangxi Province had the lowest (0.01024 ± 0.00062). Regionally, the highest *π* value was detected in Southwest China (0.01549 ± 0.00026) and the lowest was detected in Northwest China (0.01162 ± 0.00081). Regarding *Hd*, Yunnan, Guangxi, Henan, and Zhejiang Provinces showed the highest values (>0.9), while Fujian and Jiangxi Provinces had the lowest (<0.8) (Table 1). Combining the results for *Hd* and *π,* diversity was the highest in chickens from Southwest China, especially those from Yunnan Province, and the lowest in chickens from Northwest China. Interestingly, it shows near levels of *π* in both BBCs and non-BBCs, with the exception of those in Yunnan Province, where it harbors higher *π* values in BBCs than in non-BBCs (Tables 1 and S7). Estimates of genetic diversity for major haplogroups are presented in Table S8. Briefly, *π* values were the highest in haplogroups F and G in Southwest China.

**Table 1.** Genetic diversity of mtDNA D-loop in 20 Black-boned chicken breeds.

Region	Province	Breed code	*n*	*V*	*h*	*Hd* (SD)	*π* (SD)	*K*	*Fs*	*D*
Northwest China	Shaanxi	LY	16	13	5	0.825 (0.052)	0.01162 (0.00081)	6.017	3.962	2.06614
Southwest China			366	57	75	0.936 (0.007)	0.01549 (0.00026)	8.025	−24.203*	−0.15605
	Yunnan		233	50	54	0.932 (0.009)	0.01624 (0.00025)	8.415	−15.235*	0.10210
		WLS	58	32	18	0.920 (0.016)	0.01633 (0.00048)	8.457	0.162	0.85904
		TC	45	29	15	0.881 (0.032)	0.01515 (0.00074)	7.845	0.477	0.75652
		YJ	130	41	31	0.922 (0.012)	0.01537 (0.00038)	7.963	−3.354	0.17289
	Sichuan		48	24	15	0.896 (0.022)	0.01199 (0.00091)	6.211	−0.370	0.48655
		SCM	13	19	8	0.910 (0.056)	0.01312 (0.00141)	6.795	0.111	0.46782
		SCS	35	21	9	0.847 (0.033)	0.00982 (0.00136)	5.089	1.682	−0.00683
	Guizhou		85	31	24	0.899 (0.018)	0.01229 (0.00053)	6.365	−3.250	0.19785
		GZM	38	24	12	0.762 (0.068)	0.00994 (0.00149)	5.151	−0.101	−0.33428
		WM	16	17	9	0.867 (0.065)	0.01062 (0.00156)	5.500	−0.558	0.29167
		ZX	31	23	12	0.862 (0.039)	0.01243 (0.00090)	6.439	0.142	0.41568
South China	Guangxi	GX	20	19	10	0.905 (0.041)	0.01180 (0.00114)	6.100	−0.206	0.52572
Central China			102	26	26	0.913 (0.013)	0.01277 ( 0.00024)	6.604	−3.323	1.10906
	Henan	XC	20	23	14	0.958 (0.028)	0.01417 (0.00097)	7.342	−3.071	0.50957
	Hubei		36	15	9	0.848 (0.032)	0.01146 (0.00055)	5.925	2.481	2.07386
		HBS	21	14	5	0.767 (0.051)	0.00859 (0.00160)	4.448	3.596	0.51697
		YX	15	14	5	0.705 (0.112)	0.01050 (0.00211)	5.429	3.272	1.03384
	Hunan	XF	46	23	13	0.860 (0.034)	0.01199 (0.00074)	6.211	0.652	0.61315
East China			340	39	41	0.857 (0.012)	0.01217 (0.00033)	6.302	−7.157	0.09710
	Zhejiang	JS	89	32	33	0.926 (0.017)	0.01386 (0.00061)	7.180	−8.875*	0.42012
	Jiangxi		138	26	14	0.790 (0.020)	0.01024 (0.00062)	5.305	2.234	0.35364
		HY	20	21	6	0.768 (0.062)	0.01346 (0.00106)	6.974	4.220	0.67980
		SK	94	18	8	0.707 (0.029)	0.00733 (0.00063)	3.799	3.575	0.22899
		YG	24	20	7	0.775 (0.063)	0.01153 (0.00090)	5.971	2.998	0.41886
	Fujian		113	22	11	0.796 (0.024)	0.01206 (0.00041)	6.245	5.201	1.45804
		DH	40	21	10	0.855 (0.033)	0.01233 (0.00103)	6.386	2.393	0.97596
		JH	73	19	6	0.716 (0.032)	0.01132 (0.00053)	5.865	9.024	1.49907
Totals			844	76	116	0.917 (0.0049)	0.01422 (n.d.)	7.365	−23.987*	−0.69434

Breed codes: LY: Lueyang; WLS: Wuliangshan Black-boned; TC: Tengchong White; YJ: Yanjin Black-boned; SCM: Sichuan Mountain Black-boned; SCS: Sichuan Silky; GZM: Guizhou Mountain Black-boned; WM: Wumeng Black-boned; ZX: Zhuxiang; GX: Guangxi Black-boned; XC: Xichuan Black-boned; HBS: Hubei Silky black; YX: Yunxian Black-boned; XF: Xuefeng Black-boned; JS: Jiangshan Black-boned; HY: Huangyu Black-boned; SK: Silkies; YG: Yugan Black-boned; DH: Dehua Black; JH: Jinhu Black-boned. *n*: number of samples; *V*: variable sites; *h*: number of haplotypes, numbers in this bracket indicate the private haplotypes to this region or breed; *Hd*, haplotype diversity; π: nucleotide diversity; SD, standard deviation; *K*, average number of nucleotide differences; *Fs*, Fu’s *Fs* tests; *D*, Tajima’s *D* test; Statistically significant comparisons are marked by asterisks (*p* < 0.05).

### Genetic divergence

To evaluate genetic differentiation among chickens, pairwise *F*_ST_ values were computed using ARLEQUIN 3.5 according to chicken breeds and provinces. A non-metric multidimensional scaling plot was subsequently generated based on the pairwise *F*_ST_ values to ascertain population/province relationships (Figure S2). The Yunxian Black-boned breed showed the greatest differentiation from other breeds, while Silkies, Hubei Silky black, and Sichuan Silky breeds were clustered together (Figure S2A). The BBC populations from Yunnan Province were significantly divergent from those from other provinces (Figure S2B).

The AMOVA showed that variance was higher within populations than among populations within groups or among groups (Table S9). This suggests that the molecular variance mainly exists within breeds, followed by provinces, and finally regions. These results are generally in accordance with the results of *F*_ST_ analyses.

### Demographic history

Neutrality tests and a mismatch distribution analysis of BBCs and non-BBCs were performed to detect population trajectories of BBCs. As determined by Fu’s *Fs* neutrality statistics for BBCs, only chickens from Yunnan Province and Southwest China showed significant deviations from neutrality, but none of the breeds showed deviations from neutrality (Table 1). All Tajima’s *D* tests for BBCs showed no significant departure from neutrality (Table 1). In the Fu’s *Fs* neutrality tests of non-BBCs, however, departures from neutrality were detected in populations from Yunnan, Henan, and Hubei Provinces (Table S7). The mismatch distribution graph is in accord with the results of neutrality tests (Figure S3).

## Discussion

Based on the most comprehensive dataset to date, including 844 mtDNA d-loop sequences for 20 breeds from 11 provinces across China, we characterized genetic diversity and genetic differentiation in Chinese BBCs, which show interesting geographical patterns. Haplogroups A–G were disproportionately observed among the 844 chickens. Additionally, these haplogroups showed differential geographical endemicity. Common haplogroups, including A, B, and E, were found in all eleven provinces. However, haplogroup C was absent in Northwest China, haplogroup D occurred in Central and East China, while haplogroups F and G were restricted to Southwest China. Interestingly, non-BBCs in China showed a similar geographical distribution pattern to those of haplogroups F and G.

Specific mitochondrial haplogroups are widely used as candidate genetic markers to trace the demographic history of chickens (Lan et al. 2017), including chicken C1 (Huang et al. 2018a) and haplogroup D (Herrera et al. 2017; Zhang et al. 2017). Thus, the geographical distribution observed in the current study suggests that BBC breeds associated with different areas are likely to have distinct demographic histories.

To evaluate this possibility, we performed detailed genetic analyses of mtDNA D-loop sequence data for Chinese BBCs. Compared with that of other indigenous Chinese chickens, such as chicken breeds from Guangdong Province and its adjacent regions (Huang et al. 2018b), Jiangsu Province (Jia et al. 2017), East China (Jia et al. 2017), and other regions in China (Huang et al. 2018b), BBCs maintained a high level of genetic diversity. In particular, genetic diversity of BBCs from Southwest China was higher than diversity estimates for chickens from other regions, whereas BBCs from East China showed the lowest diversity. This observation may be explained by differences in traditional cultures as well as different intensities of selection. BBCs are fascinating breeds owing to their unique appearance, and local residents tend to limit crossbreeding to maintain characters. Silkies is a famous BBC breed with an atypical fluffy plumage; it was officially recognized in 1874 in North America as the Standard of Perfection (Ekarius 2007). Owing to its popularity, Silkies has been subjected to intense selection, resulting in a decline in genetic diversity (Jia et al. 2017). With a relatively large sample size, we detected few exclusive haplotypes and low genetic diversity in Silkies, confirming its breeding history.

Neutrality tests indicated that BBCs in China have not undergone rapid population expansion. The mismatch distribution graph showed that BBCs and non-BBCs share similar demographic trajectories, except for those from Henan and Hubei provinces. This finding is in accordance with the results of Huang et al. (2018a). A neighbor-joining network did not indicate BBC-specific haplogroups or major haplotypes. Consistent with the estimates of genetic diversity and genetic divergence and with husbandry reports, numerous BBC breeds endemic to China may be selected *in situ* from local domestic chickens (Weng et al. 2019).

The significant differentiation between chickens from Southwest China, particularly Yunnan Province, and those from other regions suggests that BBCs of Southwest China experienced limited gene flow with each other and with chicken from other regions, with only weak genetic selection (Miao et al. 2015). Both Fu’s *Fs* and pairwise *F*_ST_ values indicated that no breed deviates from neutrality, whereas deviations were detected at the province/region level. It is noteworthy that genetic analyses by haplogroups showed that F and G, privatized to Southwest China, have higher *π* values than those of other haplogroups, including A and C. Although previous research has reported that the chickens from Yunnan Province display high levels of genetic diversity and multiple unique mtDNA haplogroups (Liu et al. 2006; Miao et al. 2013; Huang et al. 2018a), the complex history of chicken domestication (Xiang et al. 2014; Peng et al. 2015b; Peters et al. 2015, 2016; Eda et al. 2016; Huang et al. 2018a) necessitates further research using more robust approaches and high-density markers, such as genome-wide SNPs, to establish whether the breeding center of Chinese BBCs is in Southwest China and to obtain a more in-depth understanding of BBC evolution.

## Data Availability

The mitochondrial DNA D-loop data were submitted to NCBI under the GenBank accession nos. MH923578–MH923923.
